# Association between thyroid cancer and cardiovascular disease risk: a nationwide observation study

**DOI:** 10.1038/s41598-022-22462-z

**Published:** 2022-11-02

**Authors:** Ming-Chieh Tsai, Cheng-Tzu Hsieh, Hsin-Yin Hsu, Tzu-Lin Yeh, Wen-Chung Lee, Chun-Ju Chiang, Bo-Yu Hsiao, Jing-Rong Jhuang, Wen-Hsuan Tsai, Shih-Ping Cheng, Chao-Liang Chou, Chun-Chuan Lee, Sung-Chen Liu, Po-Jung Tseng, Kuo-Liong Chien

**Affiliations:** 1 Institute of Epidemiology and Preventive Medicine, College of Public Health, National Taiwan University, Room 517, No.17, Xu-Zhou Rd., Taipei City, 10055 Taiwan; 2 Division of Endocrinology and Metabolism, Department of Internal Medicine, Taipei Mackay Memorial Hospital, No. 92, Section 2, Zhongshan North Road, Taipei City, 10449 Taiwan; 3 Department of Medicine, Mackay Medical Collage, No. 46, Sec. 3, Zhongzheng Rd., Sanzhi Dist., New Taipei City, 25245 Taiwan; 4 Department of Epidemiology, University of California, Los Angeles, USA; 5 Department of Family Medicine, Taipei MacKay Memorial Hospital, No. 92, Section 2, Zhongshan North Road, Taipei City, 10449 Taiwan; 6 Department of Family Medicine, Hsinchu MacKay Memorial Hospital, No. 690, Section 2, Guangfu Road, East District, Hsinchu City, 30071 Taiwan; 7 Division of General Surgery, Department of Surgery, Taipei Mackay Memorial Hospital, No. 92, Section 2, Zhongshan North Road, Taipei City, 10449 Taiwan; 8 Department of Neurology, Taipei Mackay Memorial Hospital, No. 92, Section 2, Zhongshan North Road, Taipei City, 10449 Taiwan; 9 Division of Cardiovascular Surgery, Department of Surgery, Hsin Chu Armed Force Hospital, Hsinchu, Taiwan; 10 Department of Internal Medicine, National Taiwan University Hospital, No. 7, Zhongshan S. Rd., Zhongzheng Dist., Taipei City, 10002 Taiwan

**Keywords:** Cancer epidemiology, Cardiology, Endocrine system and metabolic diseases

## Abstract

Treatment with levothyroxine and radioiodine contribute alternative cardiovascular function in adults with thyroid cancer. The risks of long-term cardiovascular conditions among thyroid cancer patients is unknown. This study aimed to compare the incidence of coronary heart disease (CHD), ischemic stroke (IS), and atrial fibrillation (AF) among adults with thyroid cancer with that of the general population, especially when stratified by age (< 65 and ≥ 65 years old). This observational cohort study enrolled patients between January 1, 2011 and December 31, 2016 with a follow-up until December 31, 2018. This study analyzed the data of Taiwanese thyroid cancer patients registered on the National Taiwan Cancer Registry Database, with CHD and IS. SIR models were used to evaluate the association between thyroid cancer and CHD, IS, AF, and cardiovascular disease outcome, stratified by age and sex. SIR analyses were also conducted for both sexes, age groups (< 65, ≥ 65 years), and different follow-up years. After excluding 128 individuals (< 20 years or ≥ 85 years old) and with missing index data, 4274 eligible thyroid cancer patients without CHD history, 4343 patients without IS history, and 4247 patients without AF history were included for analysis. During the median follow-up of 3.5 (1.2) years among thyroid cancer patients, the observed number of new CHD events was 70; IS, 30; and AF, 20, respectively. The SIR was significantly higher for CHD (SIR, 1.57; 95% confidence interval [CI] 1.2–1.93) among thyroid cancer patients compared with the age- and sex-specific standardized population. However, the association between thyroid cancer and the risks of IS (SIR, 0.74; 95% CI 0.47–1), cardiovascular disease (SIR, 0.88; 95% CI 0.7–1.05), and atrial fibrillation (SIR, 0.74; 95% CI 0.42–1.06) were insignificant. Moreover, stratification by age < 65 or age ≥ 65 years old and by sex for CHD suggested that the diagnosis of thyroid cancer in the young may attenuate the CHD risk (SIR, 2.08; 95% CI 1.5–2.66), and the CVD risk was constant among both men (SIR, 1.63; 95% CI 1.03–2.24) and women (SIR, 1.53; 95% CI 1.06–1.99). The patients had persistent higher CHD risk for 5 years after cancer diagnosis. Thyroid cancer survivors have a substantial CHD risk, even at long-term follow-up, especially in those patients < 65 years old. Further research on the association between thyroid cancer and CHD risk is warranted.

## Introduction

Thyroid cancer was reported to be the fifth most common cancer among women and the ninth most common cancer among both sexes in 2018. Its incidence has dramatically increased worldwide over recent decades^1^, with a 211% increase in the United States between 1975 and 2013^2^, and a 490% increase in South Korea between 1995 and 2005^3^; nevertheless, thyroid cancer-related mortality rates have not changed substantially^4^. Initial thyroid cancer treatment comprises total thyroidectomy, radioactive iodine therapy, and administration of exogenous thyroxin to facilitate thyroid stimulating hormone suppression^4^.

Radioactive iodine and thyroid stimulating hormone suppressive therapies can lead to short-term adverse events, such as thyroiditis, hypothyroidism and hyperthyroidism-related palpitations, and progressive atherosclerosis in the carotid intima^5^. Although several studies have examine the influence of these therapies on cardiac function, by measured left ventricular ejection^6^, sympathetic and vagal tone^7^, the long-term risk of coronary heart disease (CHD), ischemic stroke (IS), and atrial fibrillation (AF) in individuals with thyroid cancer, this area remains controversial. For example, some studies have demonstrated that thyroid cancer patients had higher odds (ranging from 1.05 to 1.28) of developing cardiovascular disease^8,9^. However, another study failed to find any association between thyroid cancer and the risk of cardiovascular disease^10^. Furthermore, some research found an increased risk (ranging from 1.06 to 3.95%) of AF among thyroid cancer patients^8,11^, while another study did not find any such association^12^. Using a nationwide population-based cohort, we aimed to investigate the following hypotheses: (1) CHD and IS are more frequent in thyroid cancer patients than they are in the general reference population; however, AF is as frequent in thyroid cancer patients as it is in the general reference population, and (2) the risk of CHD, IS, and cardiovascular disease among thyroid cancer patients is the same across different ages, sexes, and follow-up periods.

## Materials and methods

This retrospective cohort study used data from the National Taiwan Cancer Registry Database (NTCRD). The data analysis was conducted for the period between January 1, 2011 and December 31, 2016, and for the follow-up until December 31, 2018. The NTCRD was set up in 1979 to collect data of all confirmed cancer diagnoses among Taiwanese citizens since 2002, and it has been revised three times to date. The NTCRD records cancer characteristics including staging; first-time surgical treatment; chemo-, radiation, hormone, and target therapy; and lifestyle habits such as smoking and alcohol and betel nut consumption. Additionally, the NTCRD was merged with the National Health Insurance Research Database (NHIRD) and national death records. The NHIRD incorporated longitudinal information comprising 99% of citizens' demographic, medical, and pharmaceutical data based on the International Classification of Disease, 9th revision (ICD-9), medical procedure data, hospitalization and outpatient clinic records, and drug prescriptions. The national death records list the specific causes of death based on the ICD-9/ICD-10 codes. Additional details on the cohort protocol are available in a previously published study^13^. The protocol was reviewed and approved by the Research Ethics Committee of Mackay Memorial Hospital 20MMHIS475e. The need for informed consent was waived by MacKay Memorial Hospital Ethics committee.

For the present analysis, the baseline was defined as the day when the thyroid cancer patients received thyroidectomy in the period from January 1, 2011 to December 31, 2016. From all the patients who underwent thyroidectomy, we excluded those who were < 20 or ≥ 85 years old, had cancers other than thyroid cancer (n = 362), and had history of CHD (n = 101), IS (n = 32), or AF before the index date (n = 128) (Supplementary Fig. 1).

### Assessment of coronary heart disease, ischemic stroke, atrial fibrillation, and cardiovascular diseases

After thyroid cancer diagnosis, patients were followed up until December 31, 2018. The endpoint of primary interest among both thyroid cancer patients and the general reference population was hospitalization for CHD. This was defined as fatal and non-fatal CHD (including non-fatal myocardial infarction and coronary artery bypass graft surgery) after thyroid cancer diagnosis in the NHIRD claim records based on ICD-9 (Table 1). Fatal CHD was defined as death caused by CHD, as listed in the death certificate on the national death records. Secondary interest included IS defined by one hospitalization diagnosis, and AF defined by one hospitalization or two outpatient clinic diagnoses after thyroid cancer was diagnosed by ICD-9. The definition of cardiovascular diseases was CHD or IS.

**Table 1:** Baseline characteristics of Taiwan thyroid cancer cohort patients without coronary heart disease at baseline.

Characteristics	N	%
All	4274	100
Mean age (SD)	49	13.1
**Sex**		
Men	1041	24.4
Women	3233	75.6
**Age at diagnosis (years old)**		
20–39	1163	27.2
40–64	2658	62.2
60–84.9	453	10.6
**BMI (kg/m**^**)2**^		
< 18.5	156	3.8
18.5–23.9	1874	46
24.0–26.9	1074	26.4
≧ 27.0	971	23.8
**Urbanization**		
Urban	3267	76.4
Non-urban	1007	23.6
Current smoking or quit < 15 years	366	8.6
**Histology**		
Papillary thyroid cancer	3899	91.2
Follicular thyroid cancer	222	5.2
Medullary thyroid cancer	23	0.5
Other or missing	130	3.0
**TNM staging**		
Stage I	2865	68.6
Stage II	248	5.9
Stage III	754	18
Stage IV	312	7.5
Hypertension	1814	42.4
Diabetes	473	11.1
Hyperlipidemia	820	19.2
Aspirin used	423	9.9

### Statistical analyses

We compared the CHD incidence among Taiwanese thyroid cancer patients with that of the general population using the standardized incidence ratio (SIR), computed as the ratio of observed to expected CHD cases, stratified by age and sex. The incidences of CHD, IS, and AF were compared with the age- and sex-standardized disease incidence rates in the Taiwanese population, accounting for person-years of observation. CHD, IS, and AF incidence data from the NHIRD were used as a reference.

We computed SIRs separately among thyroid cancer patients for both sexes, each age group (20–39, 40–64, ≥ 65 years), and different follow-up periods, and compared them with that of the reference population. The cut-point age for classification as elderly was 65 years old, according to the normal age for retirement and categorization of senior citizenship in Taiwan. The changes in trends of SIRs for both sexes and for those aged < 65 or ≥ 65 years old in different follow-up years were analyzed. The incidence rates of CHD and IS among patients with all subtypes of thyroid cancer, papillary thyroid cancer (PTC), follicular thyroid cancer (FTC) or medullary thyroid cancer (MTC) were calculated according to different follow-up years. We tested whether there were different CHD or IS hazard risks between thyroid cancer subtypes by using a Cox proportional hazards model comparing CHD and IS risk between those with PTC and those with FTC. SAS version 9.4 (SAS Institute, Cary, NC, USA) and Stata version 12 (Stata Corporation, College Station, TX, USA) were used for statistical analyses.

### Ethics approval and consent to participate

The Mackay Memorial Hospital Committee Review Board approved the study protocol (IRB 20MMHIS475e approved with exempt review). The need for informed consent was waived by MacKay Memorial Hospital Ethics committee. The authors confirm that all of the research meets the ethics guidelines, including adherence to the legal requirements of the country where the study was performed.

## Results

### Sample characteristics

After excluding 128 individuals who were < 20 or ≥ 85 years old or had missing index data, 4375 thyroid cancer patients were eligible for the study. Further, 4274 patients without CHD history, 4343 patients without IS history, and 4247 patients without AF history were included for CHD, IS, and AF analysis, respectively (Supplement Fig. 1). A total of 4274 thyroid cancer patients without CHD history [mean (SD) age, 49 (13.1) years; women, 3233 (75.6%); mean (SD) follow-up, 3.5 (1.2) years; PTC, 3899 (91.2%), FTC, 222 (5.2%); and MTC, 23 (0.5%)] were analyzed for the observation CHD events. The basic characteristics of the thyroid cancer patients in the study are shown in Table 1.

### The standardized incidence ratio of coronary heart disease, ischemic stroke, cardiovascular disease, and atrial fibrillation

**Figure 1:**
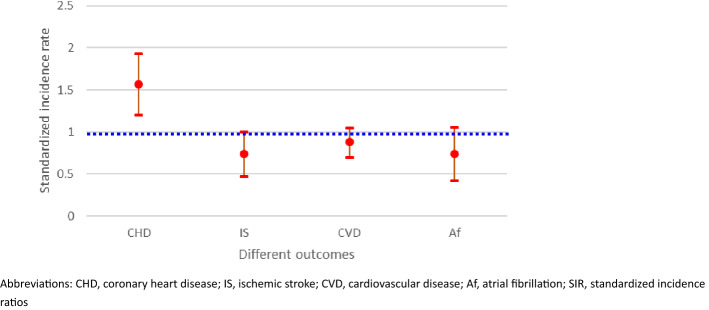
Cardiovascular and atrial fibrillation incidence, displayed as standardized incidence ratios (SIR) and 95% confidence intervals subdivided by five cardiovascular and atrial fibrillation endpoints. The blue line crossing SIR 1.0 equals the incidence rate of the general population.

The number of observed CHD events was 69, and that of expected events was 51.8 after a follow-up of 13,435 person-years. The SIR was significantly higher for CHD (SIR, 1.57; 95% confidence interval [CI] 1.2–1.93) among thyroid cancer patients compared with the age- and sex-specific standardized population (Table 2 and Fig. 1). The 30 observed events were compared with 45 expected events for IS after a follow-up of 13,730 person-years. The IS SIR was insignificant among thyroid patients (SIR, 0.74; 95% CI 0.47–1). The SIR for total CVD, estimated by dividing the 91 observed events by the 85.2 expected events, was insignificant (SIR, 0.88; 95% CI 0.7–1.05). Incident atrial fibrillation events occurred in 20 thyroid cancer patients during the 13,692 person-years follow-up. Compared with standardized general population, with 27.1 expected events, thyroid cancer patients had an insignificant risk of atrial fibrillation (SIR, 0.74; 95% CI 0.42–1.06).

**Table 2:** Cardiovascular incidence, displayed as standardized incidence ratios (SIR) and 95% confidence intervals subdivided by three cardiovascular endpoints and atrial fibrillation.

Outcome	Observed event	Expected events	Person-year	Standardized incident ratio	Lower limit	Upper limit
CHD	69	51.8	13,435	1.57	1.2	1.93
IS	30	45	13,730	0.74	0.47	1
CVD	91	85.2	13,331	0.88	0.7	1.05
Af	20	27.1	13,692	0.74	0.42	1.06

*CHD* coronary heart disease, *IS* ischemic stroke, *CVD* cardiovascular disease, *Af* atrial fibrillation, *SIR* standardized incidence ratios, *CI* confidence interval.

### The standardized incidence ratio of coronary heart disease stratified by age, sex, and follow-up year

**Figure 2:**
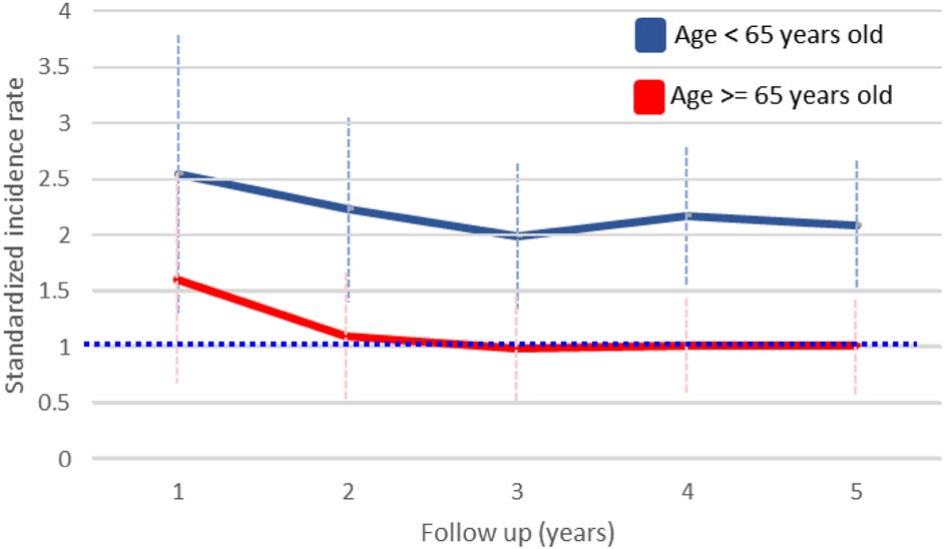
Standardized incidence for coronary heart disease at different follow up years stratified by age younger than 65 years old and older than 65.

Stratification by age (< 65 or ≥ 65 years old) for CHD suggested that the diagnosis of thyroid cancer in the young may attenuate the association between thyroid cancer and CHD risk (SIR, 2.08; 95% CI 1.5–2.66), compared with general population (Table 3). Thyroid cancer was not associated with increasing CHD risk among thyroid cancer patients diagnosed at ≥ 65 years old (SIR, 1; 95% CI 0.57–1.42). Furthermore, a positive association between thyroid cancer and CHD risk remained among both men (SIR, 1.63; 95% CI 1.03–2.24) and women (SIR, 1.53; 95% CI 1.06–1.99). Additionally, among < 65 year-olds in different follow-up years, patients were found to have significantly increasing CHD risk, persistent for 5 years after cancer diagnosis (Fig. 2 and Table 4). Conversely, similar risk trends were not detected among ≥ 65 year-old thyroid cancer patients.

**Table 3:** Standardized incidence ratio by potential explanatory factors for coronary heart disease.

Subgroup	Incidence events	SIR	95% CI	
			Lower	Upper
**All**	70	1.57	1.2	1.93
**Age at diagnosis**				
< 65	49	2.08	1.5	2.66
≥ 65	21	1	0.57	1.42
**Sex**				
*Men*	28	1.63	1.03	2.24
Men: 20–39	2	1.25	0	3.7
Men: 40–64	20	1.62	0.93	2.32
Men: ≥ 65	6	0.78	0.1	1.47
*Women*	42	1.53	1.06	1.99
Women: 20–39	1	1.8	0	5.33
Women: 40–64	26	1.59	0.98	2.2
Women: ≥ 65	15	1.01	0.5	1.53
**Follow-up (years)**				
1	27	2.05	1.28	2.83
2	42	1.65	1.15	2.15
3	53	1.5	1.09	1.9
4	67	1.6	1.22	1.99
5	70	1.57	1.2	1.93

**Table 4:** Standardized incidence for coronary heart disease at different follow up years stratified by age younger than 65 years old and older than 65.

Follow-up (years)	Age < 65 years old				Age ≥ 65 years old			
	Incidence events	SIR	95% CI		Incidence events	SIR	95% CI	
			Lower	Upper			Lower	Upper
1	16	2.54	1.3	3.79	11	1.6	0.66	2.55
2	28	2.23	1.4	3.05	14	1.09	0.52	1.66
3	36	1.99	1.34	2.64	17	0.98	0.51	1.45
4	47	2.17	1.55	2.79	20	1	0.56	1.43
5	49	2.08	1.5	2.66	21	1	0.57	1.42

### The standardized incidence ratio of ischemic stroke stratified by age, sex and follow-up years

After stratification by age (< 65 and ≥ 65 years old), elderly thyroid cancer patients were found to have a significantly lower IS risk compared with the general population (SIR, 0.6; 95% CI 0.27–0.92) (Supplemental Table 3), although the association between thyroid cancer patients at younger age and IS was not significant (SIR, 0.74; 95% CI 0.47–1). Lower numbers of IS events than expected were noted for several follow-up years among elderly thyroid cancer patients (Supplemental Table 4). Furthermore, among both women and men, a history of thyroid cancer was also noted to be associated with IS risk (Supplemental Table 3).

### The CHD and IH risk among different thyroid cancer subtypes

The CHD and IS incidence rates according to follow-up years among all thyroid cancer, PTC, and FTC patients were demonstrated. The CHD and IS incidence rates (events/1000 person-year) were 4.2 and 1.8 in the thyroid cancer group, 4.3 and 1.5 in the PTC group, and 3.4 and 3.4 in the FTC group, respectively (Supplemental Table 5 and supplemental Fig. 8). Among PTC, 65 and 24 patients subsequently developed CHD and IH, respectively, while 3 and 3 patients developed CHD and IH, respectively, among FTC patients. Compared with the PTC group as the reference group, we observed an insignificant difference in the risks of CHD and IH for FTC in the full covariate adjusted model (Supplemental Table 6).

## Discussion

The analysis of the nationally representative cohort showed that patients with thyroid cancer had a higher risk of developing CHD than the general population, although the results were not significant for IS and AF. The CHD risk was highest in young thyroid cancer patients (< 65 years of age), and this risk persisted 5 years after cancer diagnosis, while the risk was the same in thyroid cancer patients (≥ 65 years old) and the general population.

The results of the present study are in accordance with the findings from a previous study^9^ that showed that the risk of CHD events in patients with thyroid cancer is higher than that in the general population. To the best of our knowledge, no study has conducted SIR analysis for CHD risk among different age groups of thyroid cancer patients. A cohort study of 182,419 patients found increased incidence in CHD in thyroid cancer patients without a subgroup analysis by age stratification^9^. A cohort study of 3,706 thyroid cancer patients conducted a subgroup analysis of CHD risk in cancer patients < 40 and ≥ 40 years of age and a healthy control group. Although higher incidence of CHD was observed among the thyroid cancer patients, the odds were higher in the younger group (< 40 years) than those in older population (≥ 40 years) during the 1–5 year and 5–10 year follow-up periods, while it was not observed in the > 10 year follow-up^12^. Another cohort study of 6900 patients, which conducted age-based subgroup analysis of AF instead of CHD, reported that AF risk was higher among younger patients than among older patients. The SIRs of AF was 2.67 (95% CI 1.61–4.41) in patients < 45 years old and 1.03 (95% CI 0.75–1.41) in patients ≥ 75 years old^10^. These findings suggest that there is a higher long-term risk of CHD in younger patients than in older patients. In addition, our results demonstrated that the incidence of AF in thyroid cancer patients was similar to that in the general population, which is consistent with a previously published population-based study^12^ and an over-all meta-analysis result. However, in contrast to our findings, a previous study reported a significant 1.66-fold higher risk of hospitalization for AF^10,11^. The discrepancy between the results of the current study and the previous study may be attributed to several factors. First, in our study, 90% of the participants were < 65 years old, while in the previous study, one-third of the patients were > 60 years old. Young patients might be better able to tolerate the effect of subclinical thyrotoxicosis, especially after thyroid stimulating hormone suppression therapy. Second, the period of study inclusion was different in the two cohorts. In our study, thyroid cancer patients diagnosed after 2011 were included, while in the previous study, those diagnosed from 1987 to 2013 were included. Decades ago, the prevalent guidelines suggested lifelong thyroid stimulating hormone suppression treatment for all thyroid cancer patients. However, according to the current recommendations, such as the American Thyroid Association guidelines, thyroid stimulating hormone suppression treatment is not indicated for management of recurrent low-risk thyroid cancer^14^. Thus, the lower number of patients with subclinical thyrotoxicosis status could have further reduced the incidence of AF in recent years. This suggests that the recent update of recommendations on long-term AF prevention during thyroid cancer treatment may have resulted in the inconsistent results between the two studies that had different periods of inclusion.

Although the pathophysiological reasons for increased CHD incidence in thyroid cancer patients remains unclear, several factors might contribute to the higher risk. First, during thyroid cancer treatment, such as thyroidectomy, radioactive iodine therapy, and thyroid stimulating hormone suppression therapy, adverse events of hypothyroidism and hyperthyroidism status may develop. These events are involved in the development of atherosclerosis, which is directly linked to ischemic heart disease. For example, studies have reported that subclinical hyperthyroidism might contribute to increased left ventricular size^15,16^ and elevated systolic pressure, both of which reduce arterial elasticity and aggravate diastolic dysfunction^17^. Furthermore, hypercoagulability, a consequence of the pro-thrombotic effects of subclinical hyperthyroidism, can also lead to development of atherosclerosis. Second, the exposure to slowly released radioactive iodine might induce arterial atherosclerosis lasting for several years. Animal studies have demonstrated that radioactive iodine can cause atherosclerotic-like plaque formation through endothelial proliferation, chronic fibrosis of the intima and media, and occlusive changes in the vasa vasorum^18–20^. Furthermore, the intima–media thickness increased significantly after radioactive iodine ablation in hyperthyroidism patients, indicating the association of progressive atherosclerosis with radioiodine exposure^5^. However, the discrepancy in the odds for CHD and IS incidence in our study, which showed that thyroid cancer patients had a significantly higher risk of fatal and non-fatal CHD but an insignificant risk of IS, may be attributed to the different etiologies of the two conditions. In contrast to the homogenous pathophysiology of CHD, where atherosclerosis is the major cause of heart attack, the etiology of IS is more complex. Besides atherosclerosis, research has shown that cardio-embolism, small-vessel occlusion, non-atherosclerotic vasculopathies, hypercoagulable states, and hematologic disorders can all lead to IS events^21^. This heterogeneous etiology results in a more complex association between thyroid cancer and IS.

Our results support the recent recommendations^14^ and have important implications regarding cardiotoxicity prevention. From a public health perspective, considering cancer as a chronic disease with safe long-term treatment rather than a fatal disease requiring aggressive intervention may provide pragmatic benefits to cancer patients. Regarding the pathophysiological reasons for the higher standardized incidence rate of CHD among thyroid cancer patients, further evidence of the association between radioactive iodine and thyroid stimulating hormone suppression therapies and CHD should be obtained from further epidemiology studies.

### Limitations

This study has several limitations. First, the relatively short follow-up period limited our ability to detect cardiovascular events and their association with thyroid cancer. However, the inclusion period of thyroid cancer diagnosis allowed the inclusion of the recent recommendations for thyroid cancer management, rather than the recommendations prevalent decades ago. Second, although we conducted SIR analysis using age and sex stratification, residual confounding factors could have been present in our study, such as smoking and history of hypertension, diabetes, and hyperlipidemia. To deal with this, we conducted subgroup-stratified analyses of SIR and the percentage of basic characteristics in our inclusion population. Third, there was a lack of sufficient evidence for free of lead-time bias in our research. Compared with the general population, thyroid cancer patients seem to visit outpatient clinics more frequently for their follow-up care, which might result in lead-time bias. However, this potential concern may be decreasing due to the increasingly convenient and inexpensive medical health care in available in Taiwan, and the fact that that similar medical resources are available for both thyroid cancer patients and general population. Finally, the findings of this study are observational and cannot be used to establish causality; thus, further research is warranted.

## Conclusion

In conclusion, our data support the hypothesis that thyroid cancer survivors have an elevated risk of CHD compared with the general population without a history of thyroid cancers. The association was attenuated among thyroid cancer patients of a younger age, for both men and women, and was persistent after several years of follow-up. Collectively, these findings support the important role of long-term cardiotoxicity prevention during management of thyroid cancer, particular for those who are young and at a low risk of recurrence. Further research on the association between thyroid cancer and the odds of CHD, including factors such as diagnosis age, cancer characteristics, and management should be conducted.

## Supplementary Information

Supplementary Information.
