# Supplementary material for: Association between thyroid cancer and cardiovascular disease risk: a nationwide observation study
Source: Sci Rep. 2022 Nov 2;12:18438. doi: 10.1038/s41598-022-22462-z (PMC9630384; doi:10.1038/s41598-022-22462-z)
Supplement: Supplementary file 1 — Supplementary Information. [file 41598_2022_22462_MOESM1_ESM.docx]

**Supplement**

[Supplemental Table 1: International Classification of Diseases—Clinical Modification (ICD-CM) and procedure codes used to define the metabolic syndrome and clinical outcome in the study cohort 2](#_Toc74031027)

[Supplemental Table 2: Standardized incidence ratio by potential explanatory factors for ischemic stroke 6](#_Toc74031028)

[Supplemental Table 3: Standardized incidence for coronary heart disease at different follow up years stratified by men and women 7](#_Toc74031029)

[Supplemental Table 4: Standardized incidence for ischemic stroke at different follow up years stratified by men and women (A) age younger than 65 years old and older than 65 (B) 8](#_Toc74031030)

[Supplement Figure 1: The selection of study population among thyroid cancer patients in National Taiwan Cancer Registry Database cohort from 2011 to 2016 10](#_Toc74031056)

[Supplement Figure 2: Standardized incidence for coronary heart disease straited by men and women 11](#_Toc74031057)

[Supplement Figure 3: Standardized incidence for coronary heart disease at different follow up years 12](#_Toc74031058)

[Supplement Figure 4: Standardized incidence for coronary heart disease at different follow up years stratified by men and women 13](#_Toc74031059)

[Supplement Figure 5: Standardized incidence for ischemic stroke straited by men and women 15](#_Toc74031060)

[Supplement Figure 6: Standardized incidence for ischemic stroke at different follow up years 16](#_Toc74031061)

[Supplement Figure 7: Standardized incidence for ischemic stroke at different follow up years stratified by men and women (A) age younger than 65 years old and older than 65 (B) 17](#_Toc74031062)

Supplemental Table 1: International Classification of Diseases—Clinical Modification (ICD-CM) and procedure codes used to define the metabolic syndrome and clinical outcome in the study cohort

| Diagnosis | Definition | ICD-9 code | ICD-10 code | Procedure code |
| --- | --- | --- | --- | --- |
| Coronary heart disease | Either discharge diagnosis of ICD-9 or ICD-10 or procedure code | 410 | I21.01, I21.02, I21.09, I21.11, I21.19, I22.0, I22.1 | revascularization PCI (33076B, 33077B, 33078B)  CABG(68023B, 68024B, 68025B)  N26002, N26003 |
|  |  | 411 | I20.0, I24.0, I24.1, I24.8, I24.9 |  |
|  |  | 414.00 | I25.10, I25.750, I25.751, I25.758, I25.759, I25.760, I25.761, I25. 768, I25.769, I25. 811, I25.812 |  |
|  |  | 414.01 | I25.10, I25.110, I25.111, I25.118, I25.119, I25.750, I25.751, I25.758, I25.759, I25. 811 |  |
|  |  | 414.02 | I25.710, I25.711, I25.718, I25.719, I25.812, |  |
|  |  | 414.03 | I25.730, I25.731, I25.738, I25.739 |  |
|  |  | 414.04 | I25.720, I25.721, I25.728, I25.729 |  |
|  |  | 414.05 | I25.700, I25.701, I25.708, I25.709, I25.730, I25.731, I25.738, I25.739, I25.760, I25.761, I25.768, I25.769, I25.790, I25.791, I25.798, I25.799, I25.810, I25.812 |  |
|  |  | v45.81 | Z95.1 |  |
|  |  | v45.82 | Z95.5, Z98.61, Z95.8, |  |

Supplement Table 1. (Continued)

| Diagnosis | Definition | ICD-9 | ICD-10 | Procedure |
| --- | --- | --- | --- | --- |
| Ischemic Stroke | discharge diagnosis of ICD-9 or ICD-10 | 433 | 165.1,I63.02, I63.12, I65.21, I63.22, I65.1, I65.23, I65.29 , I63.031, I63.032, I63.039, I63.131, I63.132, I63.139, I63.231, I63.232, I63.239, I65.01, I65.02, I65.03, , I65.09, I63.011, I63.012, I63.019, I65.22, I63.111, I63.112, I63.119, I63.211, I63.212, I63.219, I65.8, 163.09, I63.19, I63.59, I65.9, I63.00, 163.10, I63.20, I63.29 |  |
|  |  | 434 | I66.01, I66.02, I66.03, I66.09, I66.11, I66.12, I66.13, I66.19, I66.21, I66.22, I66.23, I66.29, I66.3, I63.30, I63.311, I63.312, I63.319, I63.321, I63.322, I63.329, I63.331, I63.332, I63.339, I63.341, I63.342, I63.349, I63.39, I63.6, I66.01, I66.02, I66.03, I66.09, I66.11, I66.12, I66.13, I66.19, I66.21, I66.22, I66.23, I66.29, I66.3, I66.9, I66.40, I66.411, I66.412, I66.419, I66.421, I66.422, I66.429, I66.431, I66.432, I66.439, I66.441, I66.442, I66.449, I66.49, I66.01, I66.02, I66.03, I66.09, I66.11, I66.12, I66.13, I66.19, I66.21, I66.22, I66.23, I66.29, I66.3, I66.8, I66.9, I63.50, I63.511, I63.512, I63.519, I63.521, I63.522, I63.529, I63.531, I63.532, I63.539, I63.541, I63.542, I63.549, I63.59, I63.8, I63.9 |  |
|  |  | 435 | G45.0,G45.8,G45.1,G45.2,G46.0, G46.1, G46.2, G45.9, I67.841, I67.848 |  |
|  |  | 436 | I67.89 |  |
|  |  | 4371 | I67.81, I67.82, I67.89 |  |
|  |  | 4379 | I67.9 |  |

Supplement Table 1. (Continued)

| Diagnosis | Definition | ICD-9 code | ICD-10 code | Procedure code |
| --- | --- | --- | --- | --- |
| Atrial fibrillation | ≥2 Outpatient department or ≥1 discharge diagnosis of ICD-9 or ICD-10 | 427.31 | i48.0, i48.1, i48.2, i48.9 |  |

CABG, Coronary artery bypass graft; PCI, Percutaneous coronary intervention

Supplemental Table 2: Standardized incidence ratio by potential explanatory factors for ischemic stroke

|  |  |  |  |  |
| --- | --- | --- | --- | --- |
| Subgroup | Incidence events | SIR | 95% CI | |
|  |  |  | Lower | Upper |
| All | 30 | 0.74 | 0.47 | 1 |
| Age at diagnosis |  |  |  |  |
| < 65 | 17 | 0.89 | 0.47 | 1.32 |
| ≥ 65 | 13 | 0.6 | 0.27 | 0.92 |
| Sex |  |  |  |  |
| Men | 10 | 0.72 | 0.27 | 1.17 |
| Men: 20~39 | 0 | . | . | . |
| Men: 40~64 | 3 | 0.35 | 0 | 0.75 |
| Men: ≥ 65 | 7 | 1.08 | 0.28 | 1.89 |
| Women | 20 | 0.74 | 0.42 | 1.07 |
| Women: 20~39 | 1 | 1.66 | 0 | 4.9 |
| Women: 40~64 | 13 | 0.98 | 0.45 | 1.51 |
| Women: ≥ 65 | 6 | 0.38 | 0.08 | 0.69 |
| Follow-up (years) |  |  |  |  |
| 1 | 7 | 0.6 | 0.15 | 1.04 |
| 2 | 15 | 0.66 | 0.32 | 0.99 |
| 3 | 20 | 0.63 | 0.35 | 0.9 |
| 4 | 26 | 0.69 | 0.42 | 0.95 |
| 5 | 30 | 0.74 | 0.47 | 1 |

Supplemental Table 3: Standardized incidence for coronary heart disease at different follow up years stratified by men and women

|  |  |  |  |  |  |  |  |  |
| --- | --- | --- | --- | --- | --- | --- | --- | --- |
|  | Men | | | | Women | | | |
| Follow-up (years) | Incidence events | SIR | 95% CI | | Incidence events | SIR | 95% CI | |
|  |  |  | Lower | Upper |  |  | Lower | Upper |
| 1 | 9 | 1.8 | 0.63 | 2.98 | 18 | 2.21 | 1.19 | 3.23 |
| 2 | 15 | 1.53 | 0.76 | 2.3 | 27 | 1.73 | 1.08 | 2.38 |
| 3 | 20 | 1.47 | 0.82 | 2.11 | 33 | 1.52 | 1 | 2.03 |
| 4 | 27 | 1.68 | 1.05 | 2.31 | 40 | 1.56 | 1.07 | 2.04 |
| 5 | 28 | 1.63 | 1.03 | 2.24 | 42 | 1.53 | 1.06 | 1.99 |

Supplemental Table 4: Standardized incidence for ischemic stroke at different follow up years stratified by men and women (A) age younger than 65 years old and older than 65 (B)

| A) |  |  |  |  |  |  |  |  |
| --- | --- | --- | --- | --- | --- | --- | --- | --- |
|  | Men | | | | Women | | | |
| Follow-up (years) | Incidence events | SIR | 95% CI | | Incidence events | SIR | 95% CI | |
|  |  |  | Lower | Upper |  |  | Lower | Upper |
| 1 | ≦5 | 0.49 | 0 | 1.17 | ≦5 | 0.65 | 0.08 | 1.23 |
| 2 | 6 | 0.76 | 0.15 | 1.37 | 9 | 0.6 | 0.21 | 1 |
| 3 | 8 | 0.73 | 0.22 | 1.23 | 12 | 0.57 | 0.25 | 0.9 |
| 4 | 10 | 0.77 | 0.29 | 1.25 | 16 | 0.64 | 0.33 | 0.96 |
| 5 | 10 | 0.72 | 0.27 | 1.17 | 20 | 0.74 | 0.42 | 1.07 |

| (B) | |  |  |  |  |  |  |  |  |
| --- | --- | --- | --- | --- | --- | --- | --- | --- | --- |
|  | Age < 65 years old | | | | | Age ≥ 65 years old | | | |
| Follow-up (years) | Incidence events | | SIR | 95% CI | | Incidence events | SIR | 95% CI | |
|  |  | |  | Lower | Upper |  |  | Lower | Upper |
| 1 | ≦5 | | 0.59 | 0 | 1.26 | ≦5 | 0.6 | 0.01 | 1.19 |
| 2 | 7 | | 0.69 | 0.18 | 1.2 | 8 | 0.63 | 0.19 | 1.07 |
| 3 | 11 | | 0.75 | 0.31 | 1.2 | 9 | 0.52 | 0.18 | 0.86 |
| 4 | 15 | | 0.86 | 0.42 | 1.29 | 11 | 0.54 | 0.22 | 0.86 |
| 5 | 17 | | 0.89 | 0.47 | 1.32 | 13 | 0.6 | 0.27 | 0.92 |

Supplemental Table 5 The incidence rate according to follow-up years among all thyroid cancer patients, papillary thyroid cancer patients and follicular thyroid cancer patients; for coronary heart disease (A) for ischemic stroke (B)

(A)

| The coronary heart disease incidence rate among different thyroid cancer subtype | | | |
| --- | --- | --- | --- |
| Rate/1000 person-year | All thyroid cancer | Papillary thyroid cancer | Medullary thyroid cancer |
| Year 1 | 4.2 | 4.2 | 4.0 |
| Year 2 | 3.9 | 4.1 | 2.0 |
| Year 3 | 4.0 | 4.0 | 4.3 |
| Year 4 | 4.4 | 4.5 | 3.7 |
| Year 5 | 4.3 | 4.3 | 3.4 |
| Year 6 | 4.2 | 4.3 | 3.4 |

(B)

| The ischemic stroke incidence rate among different thyroid cancer subtype | | | |
| --- | --- | --- | --- |
|  | All thyroid cancer | Papillary thyroid cancer | Medullary thyroid cancer |
| Year 1 | 1.9 | 1.4 | 8.0 |
| Year 2 | 1.7 | 1.3 | 6.0 |
| Year 3 | 1.6 | 1.3 | 4.3 |
| Year 4 | 1.7 | 1.5 | 3.7 |
| Year 5 | 1.8 | 1.6 | 3.4 |
| Year 6 | 1.8 | 1.5 | 3.4 |

Supplemental Table 6 The incidence cases, follow-up person-years, and rates of cardiovascular disease and ischemic stroke events, hazard ratios, and 95% confidence intervals specified according to papillary thyroid cancer and follicular thyroid cancer (Note: Model 1: adjusted for age and sex; Model 2: Model 1, additionally urbanization, BMI and smoke; Model 3: Model 2, additionally stage, HTN, DM, hyperlipidemia and aspirin); For coronary artery disease (A) ischemic stroke (B)

(A)

| The CHD risk comparison between different thyroid cancer subtype | | |
| --- | --- | --- |
|  | Papillary thyroid cancer (n= 3899) | Medullary thyroid cancer (n=222) |
| Cases | 65 | 3 |
| Pearson-year | 15281.1 | 891.3 |
| Rates/1000 py | 4.25 | 3.37 |
|  | HR | HR |
| Univariate | 1 | 0.80 (0.25-2.54) |
| Model 1 | 1 | 0.68 (0.21-2.18) |
| Model 2 | 1 | 0.78 (0.24-2.50) |
| Model 3 | 1 | 0.93 (0.28-3.09) |

(B)

| The IS risk comparison between different thyroid cancer subtype | | |
| --- | --- | --- |
|  | Papillary thyroid cancer (n= 3899) | Medullary thyroid cancer (n=222) |
| Cases | 24 | 3 |
| Pearson-year | 15628.0 | 893.8 |
| Rates/1000 py | 1.54 | 3.36 |
|  | HR | HR |
| Univariate | 1 | 2.17 (0.66-7.22) |
| Model 1 | 1 | 1.56 (0.46-5.26) |
| Model 2 | 1 | 1.60 (0.47-5.46) |
| Model 3 | 1 | 1.43 (0.39-5.24) |

Supplement Figure

Supplement Figure 1: The selection of study population among thyroid cancer patients in National Taiwan Cancer Registry Database cohort from 2011 to 2016


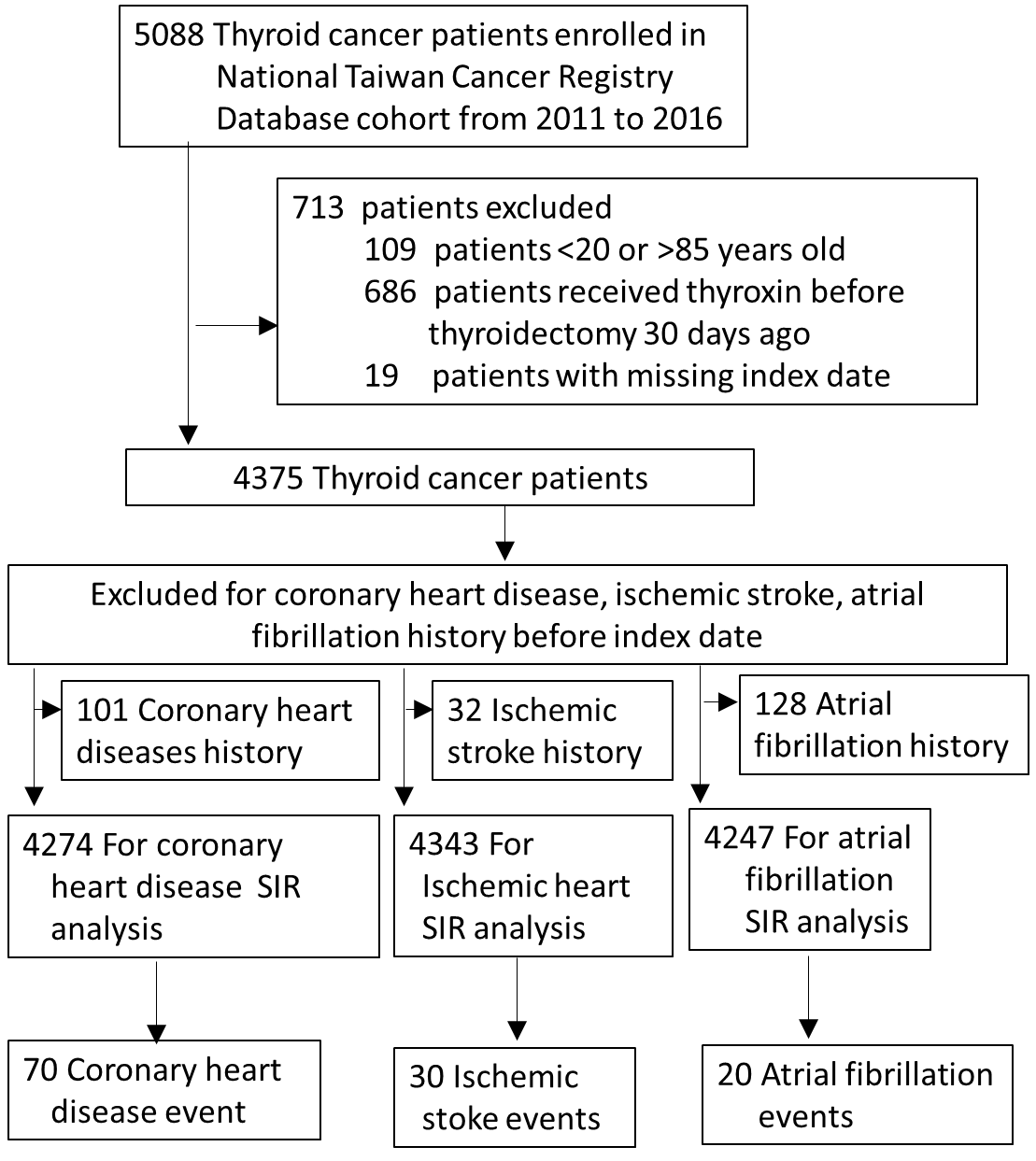


Abbreviations: SIR, standardized incidence ratios;

Supplement Figure 2: Standardized incidence for coronary heart disease straited by men and women


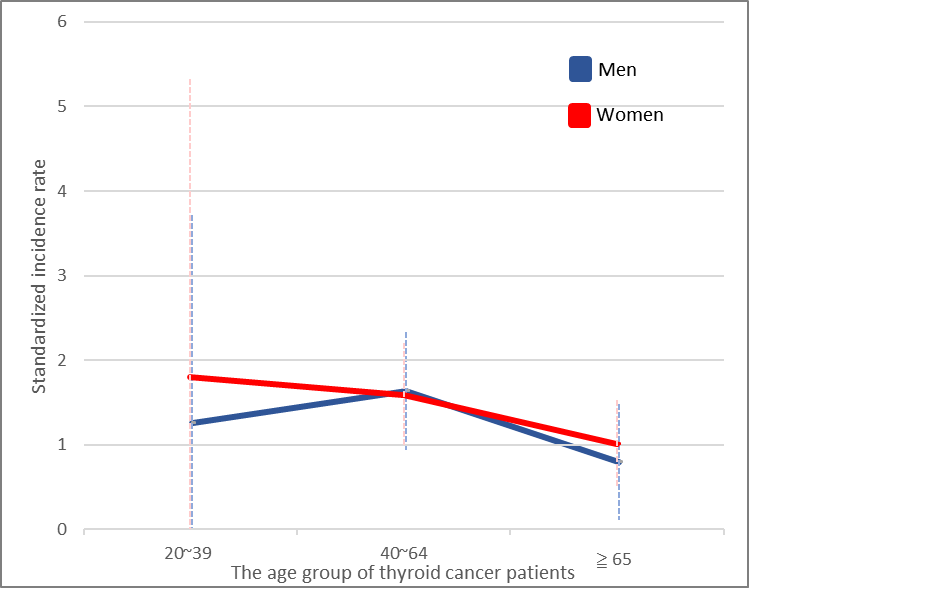


Supplement Figure 3: Standardized incidence for coronary heart disease at different follow up years


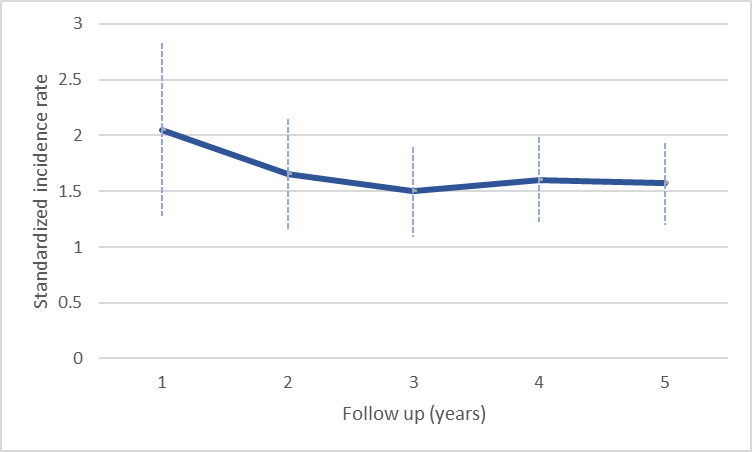
)

Supplement Figure 4: Standardized incidence for coronary heart disease at different follow up years stratified by men and women


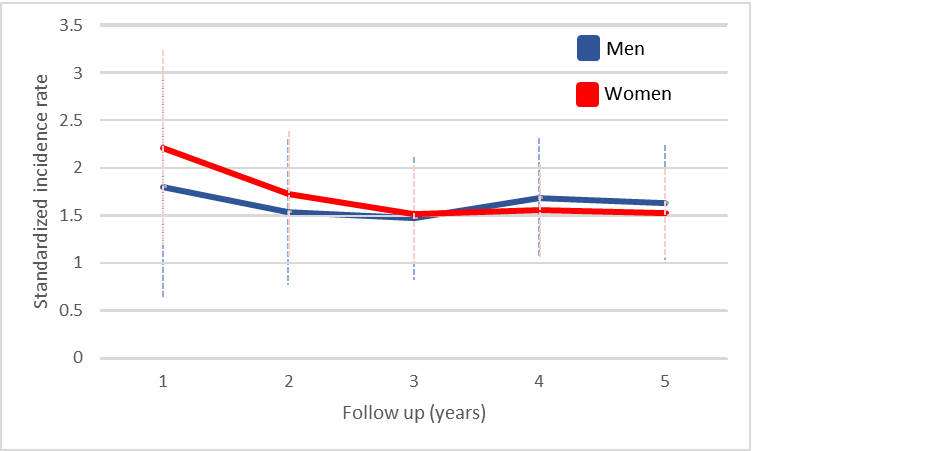


Supplement Figure 5: Standardized incidence for ischemic stroke straited by men and women


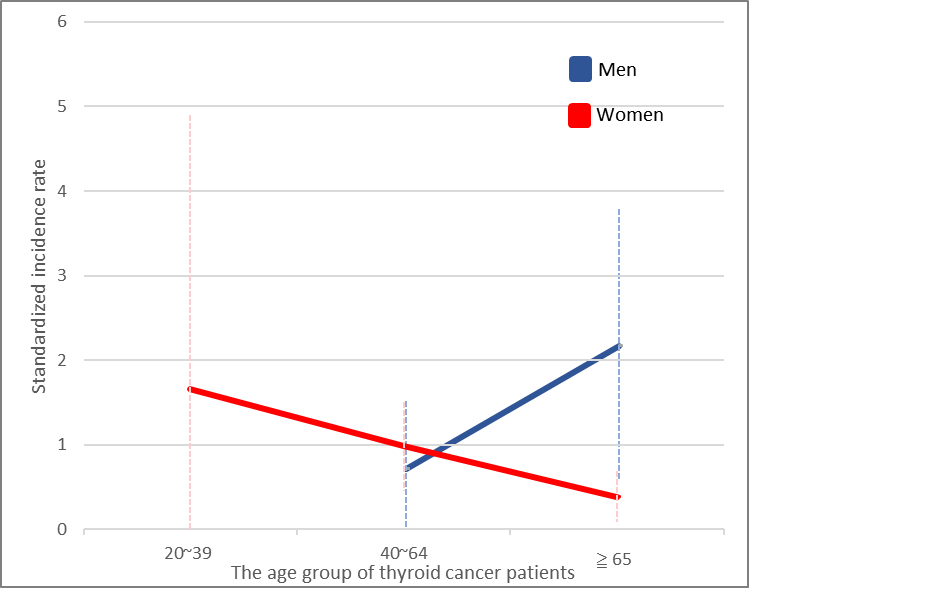


Supplement Figure 6: Standardized incidence for ischemic stroke at different follow up years


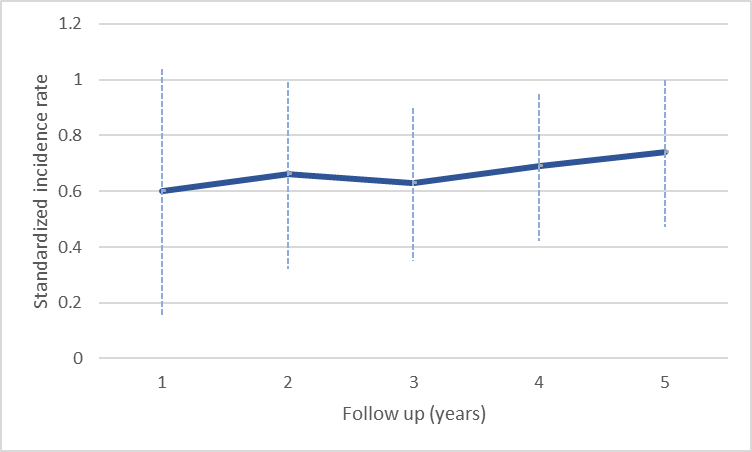


Supplement Figure 7: Standardized incidence for ischemic stroke at different follow up years stratified by men and women (A) age younger than 65 years old and older than 65 (B)

(A)


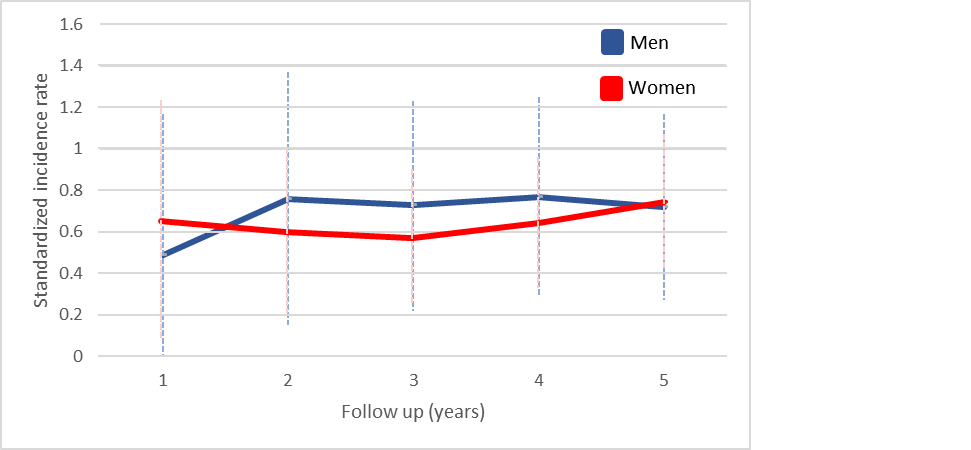


(B)
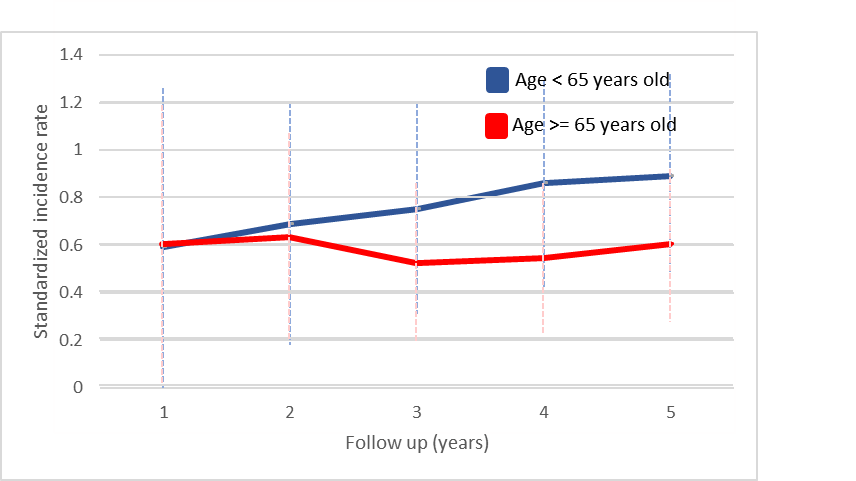


Supplement Figure 8. The incidence rate according to follow-up years among all thyroid cancer patients, papillary thyroid cancer patients and follicular thyroid cancer patients; for coronary heart disease (A) for ischemic stroke (B)

(A)


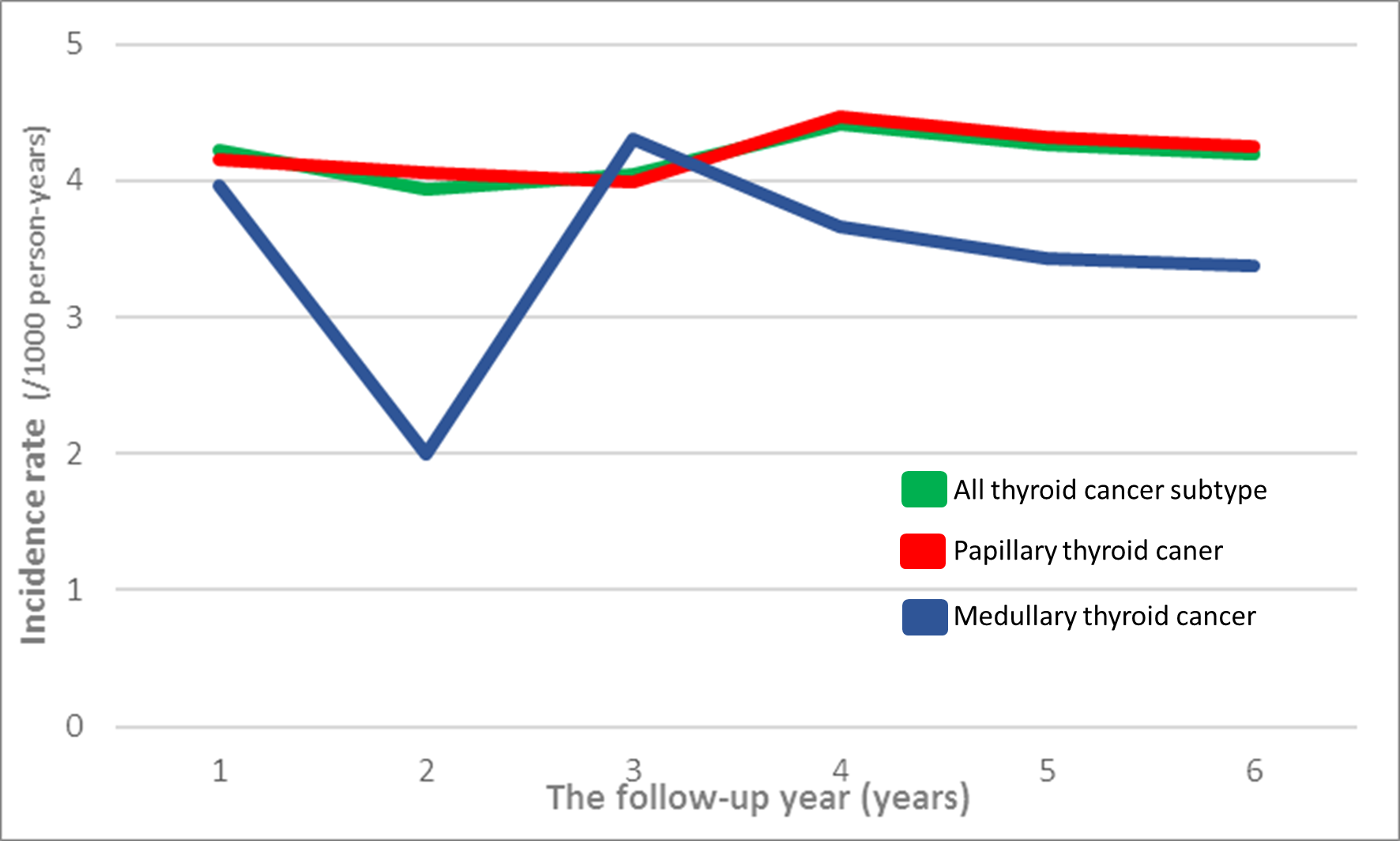


(B)


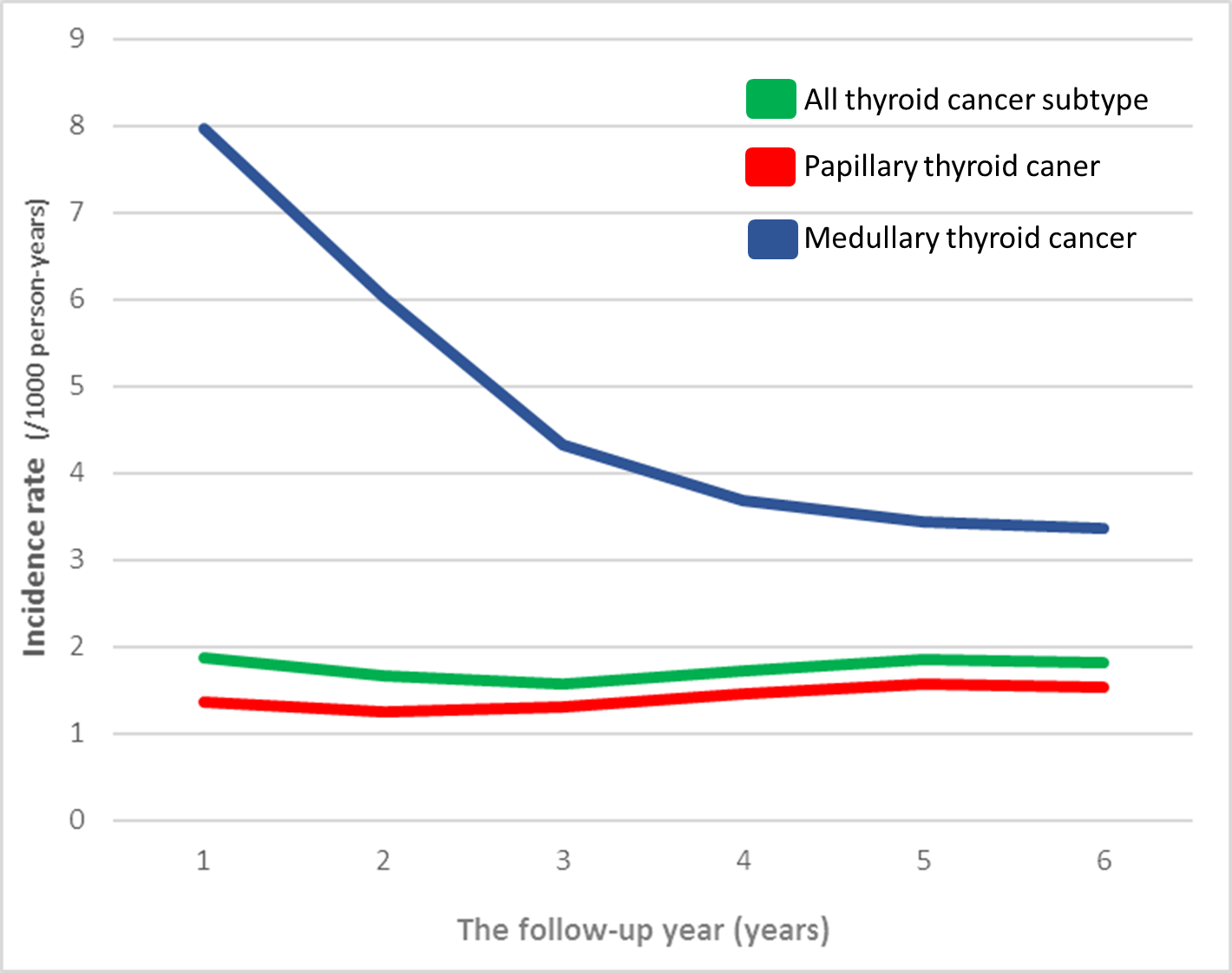
\\
